# Consumer Use of “Dr Google”: A Survey on Health Information-Seeking Behaviors and Navigational Needs

**DOI:** 10.2196/jmir.4345

**Published:** 2015-12-29

**Authors:** Kenneth Lee, Kreshnik Hoti, Jeffery David Hughes, Lynne M Emmerton

**Affiliations:** ^1^ Curtin University School of Pharmacy Curtin University Perth Australia

**Keywords:** online, health information, health literacy, patient activation, information seeking, information needs, Internet, chronic disease, patients, survey

## Abstract

**Background:**

The Internet provides a platform to access health information and support self-management by consumers with chronic health conditions. Despite recognized barriers to accessing Web-based health information, there is a lack of research quantitatively exploring whether consumers report difficulty finding desired health information on the Internet and whether these consumers would like assistance (ie, navigational needs). Understanding navigational needs can provide a basis for interventions guiding consumers to quality Web-based health resources.

**Objective:**

We aimed to (1) estimate the proportion of consumers with navigational needs among seekers of Web-based health information with chronic health conditions, (2) describe Web-based health information-seeking behaviors, level of patient activation, and level of eHealth literacy among consumers with navigational needs, and (3) explore variables predicting navigational needs.

**Methods:**

A questionnaire was developed based on findings from a qualitative study on Web-based health information-seeking behaviors and navigational needs. This questionnaire also incorporated the eHealth Literacy Scale (eHEALS; a measure of self-perceived eHealth literacy) and PAM-13 (a measure of patient activation). The target population was consumers of Web-based health information with chronic health conditions. We surveyed a sample of 400 Australian adults, with recruitment coordinated by Qualtrics. This sample size was required to estimate the proportion of consumers identified with navigational needs with a precision of 4.9% either side of the true population value, with 95% confidence. A subsample was invited to retake the survey after 2 weeks to assess the test-retest reliability of the eHEALS and PAM-13.

**Results:**

Of 514 individuals who met our eligibility criteria, 400 (77.8%) completed the questionnaire and 43 participants completed the retest. Approximately half (51.3%; 95% CI 46.4-56.2) of the population was identified with navigational needs. Participants with navigational needs appeared to look for more types of health information on the Internet and from a greater variety of information sources compared to participants without navigational needs. However, participants with navigational needs were significantly less likely to have high levels of eHealth literacy (adjusted odds ratio=0.83, 95% CI 0.78-0.89, *P*<.001). Age was also a significant predictor (*P*=.02).

**Conclusions:**

Approximately half of the population of consumers of Web-based health information with chronic health conditions would benefit from support in finding health information on the Internet. Despite the popularity of the Internet as a source of health information, further work is recommended to maximize its potential as a tool to assist self-management in consumers with chronic health conditions.

## Introduction

The Internet offers a wealth of information on numerous topics. Its pervasiveness in everyday life means it is a common source of information for many consumers [1]. Many consumers use it to obtain health-related information [2-5]. Accordingly, a number of studies have examined the role of the Internet in health care and its influence on the traditional relationship between consumers and their health professionals [6-10]. Traditionally, health professionals have been the primary source of health information, providing information through patient education [11]. Consumers are now afforded greater access to information, have greater potential to be more informed, and are able to play a greater role in caring for their health [11].

Consumers also play an important role in health care, particularly given a trend towards greater burden of chronic health conditions [12]. Such conditions often require daily self-management. In Australia, annual expenditure on chronic health conditions is estimated at AUD $11.0 billion [13]. Internationally, a number of chronic health conditions have been listed in the top 10 leading causes of mortality [14]. Consequently, initiatives should focus on supporting consumers with chronic health conditions to better manage their conditions.

The popularity of the Internet for health-related purposes enables its use to support self-management. Indeed, numerous studies have examined the popularity of Internet use as a source of health information [2,3,5,15-17]. In the United States, 80% of Internet users use it for health information [3]. It appears that the use of the Internet for health information is more popular in Internet users with chronic health conditions or disabilities compared to Internet users without chronic health conditions or disabilities [18]. While fewer data are available within the Australian context, a 2010 study [5] suggested that almost 80% of Internet users in Australia access the Internet for health information. Despite the popularity of its use for health information, a number of studies have identified barriers to accessing Web-based health information [19-22]. The volume of health information available on the Internet [19-22], the abundance of poor quality information [19,20,23], and the lack of strict publishing guidelines [19] are some examples. Furthermore, a 2001 review on consumers’ Web-based health-information seeking identified factors contributing to potential misinformation and subsequent potential for harm if consumers were to access and act upon misleading information [19]. Hence, there is a need to better understand consumers’ Web-based health information-seeking behaviors (HISB) to better support consumers in their self-management.

Numerous studies have explored the characteristics of consumers’ Web-based HISB [3-5,23-44]. However, within the context of consumers with chronic health conditions, the majority of studies appear to focus on specific chronic health conditions [24,26,28-32,34-38], age [33,41], or ethnic groups [40], or they involve general populations that include consumers without chronic health conditions [3-5,23,25,27,39]. The applicability of findings from such studies to other populations may be limited. We believe that exploration of Web-based HISB in a population of health information consumers with a variety of chronic health conditions can facilitate identification of general characteristics or trends of Web-based HISB; such characteristics can then be compared to existing and future studies that focus on specific populations.

A qualitative study was recently conducted using consumers of Web-based health information who identified as having one or more chronic health conditions [20]. This study explored the Web-based HISB of its participants and identified a number of potentially related characteristics. However, the applicability of these characteristics to a wider population is unknown. While a large-scale quantitative study has explored the characteristics of consumers with chronic health conditions and the proportion of Internet and non-Internet users [42], no large-scale quantitative studies examine the breadth of HISB characteristics reported by consumers in the aforementioned qualitative study [20]. For example, previous studies have examined characteristics such as the frequency of Internet use for health-related purposes [45] and consumers’ experiences with locating Web-based health information [46]. Within the context of health information consumers with a variety of chronic health conditions, characteristics of Web-based HISB such as the types of health information sought on the Internet and reasons for seeking Web-based health information have yet to be quantitatively determined.

Related to Web-based HISB, as identified by [20], are the concepts of health literacy, eHealth literacy, and patient activation. Numerous studies have identified health literacy [47-51] and eHealth literacy [52,53] as important skills in locating, accessing, and utilizing quality health information for health care management. Patient activation is defined as patients’ belief that they “have important roles to play in self-managing care, collaborating with providers, and maintaining their health. They know how to manage their condition and maintain functioning and prevent health declines; and they have the skills and behavioral repertoire to manage their condition, collaborate with their health providers, maintain their health functioning, and access appropriate and high-quality care” [54]. Some evidence supports a statistically significant relationship between health literacy and patient activation [55,56]. However, to our knowledge, no study to date has examined the relationship between eHealth literacy and patient activation.

Despite the aforementioned barriers to acquiring desired Web-based health information, health information seeking using the Internet remains a prevalent activity. Thus, beyond understanding consumers’ Web-based HISB, eHealth literacy, and patient activation, researchers have not yet explored whether consumers have difficulty finding, and indicate a desire for support to find, Web-based health information (ie, navigational needs). While findings from a qualitative study suggest a potential need for support interventions among consumers [20], the applicability of this finding to a wider population has yet to be determined. Furthermore, there are no studies examining potential determinants or predictors of navigational needs. Once an understanding of navigational needs and an estimate of the proportion of the population with navigational needs is ascertained, future studies can then explore consumers’ preferences for support interventions within and between various populations, such as populations with specific chronic health conditions, which better support consumers in their self-management.

Thus, this study aims to address the following objectives: (1) estimate the proportion of consumers with navigational needs among consumers of Web-based health information living with chronic health conditions, (2) describe the following characteristics of consumers with navigational needs: Web-based HISB, patient activation, and eHealth literacy, and (3) explore variables predicting navigational needs of these consumers.

## Methods

### Overview

A Web-based questionnaire was developed via the Qualtrics platform to identify the proportion of consumers with navigational needs and to explore their demographics, Web-based HISB, eHealth literacy, and patient activation.

Ethical approval for this study was granted by the Curtin University Human Research Ethics Committee (HR06/2013).

### Participants and Recruitment

The target population for this study was adult Web-based health information consumers with chronic health conditions residing in Australia. Participants were included in this study if they consented to the study and indicated they met the following criteria: (1) able to easily read and write in English, (2) aged 18 years or older, (3) use of the Internet to find information about their health, and (4) have at least one chronic health condition.

Recruitment was conducted by Qualtrics through their partnership with a Web-based survey research company, ResearchNow, which hosts a large diverse pool of participants and has the ability to select representative samples meeting specified eligibility criteria [57].

### Sample Size

The sample size was determined using conservative parameters for prevalence studies [58]—our focus for prevalence estimation being the proportion of the target population with navigational needs (Objective 1). In the absence of literature reporting this prevalence, we used the following parameters: expected population proportion of 50%, 95% confidence interval, and a level of precision of estimate within 5% either side of the true population proportion. These parameters indicated a required sample size of 385 participants [58]. To account for potential invalid responses, the required sample was increased to 400 participants (a level of precision of 4.9% either side). This sample size was also deemed adequate to conduct descriptive and inferential statistical analyses to address the other objectives. The research company was contracted to meet the quota of 400 submitted questionnaires.

### Questionnaire Development

#### Initial Questionnaire Construction

Questions and response items pertaining to navigational needs and Web-based HISB were predominantly drawn from interview questions and participant responses from a qualitative study [20] of health consumers with chronic health conditions who used the Internet. To ensure that questions asked verbally in the aforementioned qualitative study [20] were suitable for a written questionnaire, the wording of the interview questions was modified by the primary researcher with review from the other researchers on the research team. Similarly, decisions for choosing which interview questions were to be included as survey questions were made by the primary researcher in collaboration with the research team. Further items were added to supplement these questions and facilitate statistical analysis after discussion with all authors. Question types were a mix of 5-point Likert-type scales and multiple-response, multiple-choice options. Where relevant, multiple-choice items facilitated “other” responses to be typed and later manually coded for analysis. To mitigate the potential for selection bias within questions, the order of response items within each multiple-choice question was randomized where appropriate [59]. To reduce the number of questions and therefore respondent fatigue, adaptive questioning was used [59].

The eHealth Literacy Scale (eHEALS), a measure of perceived eHealth literacy [60], and PAM-13 [61], a measure of patient activation, were used to assess eHealth literacy and patient activation, respectively. Both of these scales had been assessed for validity and reliability [54,60-68] and were incorporated with permission from their respective authors/licensors.

#### Pilot Test

A target of 40 completed responses (10% of the final sample) was used to pilot test the questionnaire. Participants recruited for this stage were to meet the same eligibility criteria as our test sample and were recruited by Qualtrics via ResearchNow. Participants from the pilot sample were excluded from participation in the test sample to mitigate response bias.

The purposes of pilot testing were to assess comprehension of questions and response items and to examine questions with invalid or poor responses. Participants were encouraged, in space provided after each question, to provide comments regarding the comprehensibility of questions and response items.

#### Questionnaire Refinement

Based on participant feedback in the pilot test, a number of amendments were made to the wording of questions and response items, along with presentation of the questions for completion in Web-based format. First, the questionnaire enabled “attention-filter” questions; thus, response items were added to identify invalid responses (eg, “I am paying attention; please select ‘disagree’ for this line”). Three attention filters were included in this questionnaire: two questions instructed participants to select a certain option, and one response item instructed participants not to select the item. These attention filters were inserted into parts of the questionnaire that required longer attention spans (eg, long questions or questions with numerous response items). Second, wording of questions with lower response rates were revised, and these questions were marked as forced responses where possible to facilitate statistical analysis. To ensure participants were permitted to respond with “not applicable” for forced response questions, an “Other” option was provided wherever possible, with free-text space to explain their situation. Third, the mean survey completion time from the pilot test was relayed to Qualtrics to determine a “duration filter” for the test sample. The time parameter for the duration filter was calculated to be one-third of the mean pilot questionnaire completion time, as recommended by Qualtrics, and excluded participants who completed the questionnaire in a shorter-than-expected time. All questions and response items were examined by the research team to ensure readability and face validity prior to survey administration.

A Flesch-Kincaid Grade Level test [69] was conducted to test the readability of the questionnaire, including the informed consent and eligibility screening page, to compare to participants’ reported level of education.

#### Reliability Testing

A subset of 48 participants (approximately 10%, allowing extra in the case of delays in acceptance or questionnaire completion) was invited 2 weeks after completion of the questionnaire to retake the questionnaire, to confirm the test-retest reliability of the eHEALS and PAM-13 against reported values.

### Analysis

#### Overview

All statistical analyses were conducted using SPSS version 21. Descriptive statistics were used to address Objective 1. Descriptive statistics, Pearson correlation, and multivariate linear regression were utilized to address Objective 2. Bivariate and multivariate binary logistic regressions were conducted to address Objective 3. Scores for the eHEALS and PAM-13 were calculated as per the authors’ instructions and were used in the regression modeling (Objectives 2 and 3).

All variables to be tested in the regression analyses were entered via a forced-entry method, as this method is more stable against random variation in the data, compared to other methods such as stepwise methods [70]. Demographic variables of age, sex, and level of education were entered alongside the other test variables, as these variables have been identified as potential contributors to the usage of Web-based health information [40,71]. The demographic variable examining residence in major cities or rural areas was also included in the regression model, as rurality has been identified as a potential barrier to Internet access [5]. Given the categorical nature of our demographic variables, categories with low or zero frequencies were aggregated with other categories, where logical, to allow for valid statistical conclusions. To illustrate, for the “age” variable (see Table 1), less than 1% of participants indicated that they were above the 55-64 years age category; the categories 65-74, 75-84, and 85+ were therefore combined with the 55-64 age category and relabeled as 55+ for inferential statistical analysis. Similarly, for the remoteness of residence variable (see Table 1), few participants indicated that they reside in remote areas; this category was aggregated with rural or regional areas to allow for comparison between major city areas versus rural/regional/remote. Such decisions for aggregating categories were made by the primary researcher in discussion with all other researchers within the research team. The level of significance (alpha) was set at *P*<.05.

**Table 1:** Demographic descriptors of respondents (N=400).

Category		No navigational needs (N=195), n (%)	Navigational needs (N=205), n (%)	Total, n (%)
**Sex**				
	Male	73 (37.4)	82 (40.0)	155 (38.8)
	Female	122 (62.6)	123 (60.0)	245 (61.3)
**Age group (years)**				
	18-24	22 (11.3)	22 (10.7)	44 (11.0)
	25-34	49 (25.1)	71 (34.6)	120 (30.0)
	35-44	35 (17.9)	36 (17.6)	71 (17.8)
	45-54	52 (26.7)	30 (14.6)	82 (20.5)
	55-64	36 (18.5)	44 (21.5)	80 (20.0)
	65-74	1 (0.5)	2 (1.0)	3 (0.8)
	75-84	0 (0.0)	0 (0.0)	0 (0.0)
	≥85	0 (0.0)	0 (0.0)	0 (0.0)
**Level of formal education**				
	No formal education	0 (0.0)	0 (0.0)	0 (0.0)
	Primary school	2 (1.0)	0 (0.0)	2 (0.5)
	Junior high school	21 (10.8)	13 (6.3)	34 (8.5)
	Senior high school	38 (19.5)	47 (22.9)	85 (21.3)
	TAFE or technical college	53 (27.2)	62 (30.2)	115 (28.8)
	University	81 (41.5)	83 (40.5)	164 (41.0)
**Remoteness of residence**				
	Major city area	122 (62.6)	144 (70.2)	266 (66.5)
	Rural or regional area	69 (35.4)	61 (29.8)	130 (32.5)
	Remote area	4 (2.1)	0 (0.0)	4 (1.0)

#### Navigational Needs

The term “navigational needs” has been used above and refers to individuals who report having difficulty finding, and would like support in locating, desired Web-based health information. As no objective measure of navigational needs is available in the literature, we operationally defined the term as individuals who identified that they at least “sometimes” have difficulty locating desired Web-based health information (Criterion 1) and indicated that they would like help locating desired Web-based health information (Criterion 2).

These participants were considered a subset of the total respondents for the purposes of data analysis and were descriptively compared (Objectives 1 and 2). For Objective 3, this subset was compared to the remainder of the sample using binary logistic regression to determine predictors of navigational needs.

#### Reliability Tests

Statistical procedures to test the reliability of the eHEALS and PAM-13 [60,62,64-68] were replicated in our study (Objective 2, patient activation and eHealth literacy). These tests included internal consistency (Cronbach alpha) and intraclass correlation coefficient (ICC). ICC was assessed via a two-way mixed effects model [72] using an absolute agreement definition, ICC (3,1). This decision was made given the self-reported nature of our questionnaire and our intent to assess the agreement of participant responses to both PAM-13 and eHEALS between test and retest. Results from each of these tests were considered alongside relevant guidelines to assist interpretation [72,73].

## Results

### Summary

The survey was conducted during May 2014. In order to obtain our target of 400 submitted questionnaires, a total of 1104 individuals were invited by ResearchNow from their diverse participant pool. Of these 1104 individuals, 1027 agreed to participate (93.03% consent). Of the 1027 individuals, 514 individuals (50.05%) met our eligibility criteria, and 400 (77.82%) completed the questionnaire.

In the retest sample 2 weeks post-completion, 47 of the 48 participants contacted agreed to participate again (98% consent). Of these, 43 completed the questionnaire a second time (91% completion).

The Flesch-Kincaid Grade Level for our questionnaire, including the informed consent and eligibility screening questions, resulted in a readability score of 8.0.

### Proportion of Consumers With Navigational Needs

As established above, participants were operationally defined as having navigational needs if they met both Criterions 1 and 2. To assess Criterion 1, participants were asked to rate, on a 5-point Likert-type scale (Never, Rarely, Sometimes, Most of the Time, Always), how often they have difficulty finding desired Web-based health information. A total of 216 participants (54.0%) indicated that they experienced difficulty at least sometimes, thereby meeting Criterion 1.

To assess Criterion 2, participants indicated whether they would like help finding desired Web-based health information. A total of 365 participants (91.3%) met this criterion.

A total of 205 participants (51.3%) met both Criteria 1 and 2 for navigational needs. The estimated proportion of consumers with navigational needs among consumers of Web-based health information living with chronic health conditions was thus estimated at 51.3% (95% CI 46.4%-56.2%).

### Demographic Characteristics

Of the 400 participants, 61.3% were female (245/400), 41.0% reported having a university-level of education (164/400), and 66.5% (266/400) reported being located in major city areas within Australia (see Table 1). Descriptive comparisons of the demographics of participants with and without navigational needs are included in Table 1; significance testing of these comparisons as potential predictors of navigational needs is illustrated later. Overall, demographic characteristics between participants with and without navigational needs appear comparable (see Table 1). Noteworthy exceptions include a higher proportion of participants with navigational needs who were aged 25-34 years old compared to participants without navigational needs (34.6% vs 25.1%), and a lower proportion of participants with navigational needs who were aged 45-54 years old compared to participants without navigational needs (14.6% vs 26.7%). Reported chronic health conditions varied widely, with conditions involving the major organs most prevalent (see Multimedia Appendix 1).

### Web-Based Health Information-Seeking Behaviors

Descriptive comparisons of the Web-based HISB between participants with and without navigational needs are provided in Tables 2-4 (as well as Multimedia Appendices 2-4). Significance testing was not performed, as multiple-response items did not allow variables to be analyzed independently.

The categories of health information reportedly sought varied considerably; however, participants with navigational needs appeared to look for more types of health information compared to participants without navigational needs (see Multimedia Appendix 2). Similarly, when comparing participants with and without navigational needs, participants with navigational needs appeared to use more sources of Web-based health information (see Multimedia Appendix 3).

Most commonly, participants sought information on the Internet to be more informed and engaged in their self-care (see Table 2). In comparing participants with and without navigational needs, we found that participants with navigational needs appear to seek Web-based health information because they are less satisfied with their health professionals, but less interested in wanting to manage their own conditions (see Table 2). However, more participants with navigational needs appeared to act on the acquired health information compared to participants without navigational needs (see Multimedia Appendix 4).

**Table 2:** Why Web-based health information is sought (N=400).

Reason for seeking Web-based health information	No navigational needs (N=195), n (%)^a^	Navigational needs (N=205), n (%)^a^	Total, n (%)^a^
I want to be more informed.	155 (79.5)	169 (82.4)	324 (81.0)
I want to help manage my own condition.	143 (73.3)	127 (62.0)	270 (67.5)
I want to clarify information that has been given to me by a health professional.	114 (58.5)	109 (53.2)	223 (55.8)
Just out of interest.	105 (53.8)	107 (52.2)	212 (53.0)
I want to check information that was discussed during a consultation with a health professional.	89 (45.6)	109 (53.2)	198 (49.5)
I want to look for alternative or additional treatment options.	94 (48.2)	98 (47.8)	192 (48.0)
I want to have information to read.	91 (46.7)	93 (45.4)	184 (46.0)
I find there is limited time during a consultation with a health professional.	48 (24.6)	69 (33.7)	117 (29.3)
I am not provided with enough information during a consultation with a health professional.	38 (19.5)	61 (29.8)	99 (24.8)
I disagree with certain points made by a health professional.	17 (8.7)	24 (11.7)	41 (10.3)
Other	12 (6.2)	11 (5.4)	23 (5.8)

^a^Respondents could select multiple options; percentages do not total 100%.

Most of the participants, 94.5% (378/400) reported that they discussed health information sourced on the Internet with health professionals at least some of the time. Reasons for this behavior are suggestive of seeking professional opinion, along with a desire to engage further in self-management (see Table 3). Such reasons for discussing Web-based health information with health professionals appear comparable between participants with and without navigational needs. A notable exception is that a greater proportion of participants with navigational needs have discussions with health professionals to “find out more information” compared to participants without navigational needs.

**Table 3:** Reasons why health information obtained on the Internet is discussed with health professionals (N=378).

Consultation with health professionals	No navigational needs (N=181), n (%)^a^	Navigational needs (N=197), n (%)^a^	Total, n (%)^a^
I want to get the health professional’s opinion on information that I found on the Internet.	123 (68.0)	130 (66.0)	253 (66.9)
I want to find out more information.	98 (54.1)	121 (61.4)	219 (57.9)
I want to be in control of the management of my health condition(s).	95 (52.5)	101 (51.3)	196 (51.9)
I trust the health professional.	81 (44.8)	81 (41.1)	162 (42.9)
I want to discuss alternative treatments, tests, or procedures.	79 (43.6)	74 (37.6)	153 (40.5)
I want to clarify information that was unclear on the website(s) that I visited.	67 (37.0)	80 (40.6)	147 (38.9)
Other	3 (1.7)	5 (2.5)	8 (2.1)

^a^Respondents could select multiple options; percentages do not total 100%.

Similarly, 98.8% (395/400) of the participants reported that they do not discuss health information sourced from the Internet with health professionals at least some of the time. Common reasons reported for not always discussing Web-based health information with health professionals relate to not wanting to embarrass oneself in front of health professionals and a belief that health professionals do not have the time to discuss health information sought on the Internet (see Table 4).

**Table 4:** Reasons why health information obtained from the Internet may not be discussed with health professionals (N=395).

Reason for not discussing with health professionals	No navigational needs (N=193), n (%)^a^	Navigational needs (N=202), n (%)^a^	Total, n (%)^a^
I do not want to embarrass myself in front of my health professional.	58 (30.1)	75 (37.1)	133 (33.7)
I do not think that health professionals have enough time to discuss what I find on the Internet.	53 (27.5)	71 (35.1)	124 (31.4)
I feel that I have enough information already.	64 (33.2)	59 (29.2)	123 (31.1)
I do not want to upset my health professional.	35 (18.1)	52 (25.7)	87 (22.0)
Other	37 (19.2)	23 (11.4)	60 (15.2)

^a^Respondents could select multiple options; percentages do not total 100%.

### Patient Activation and eHealth Literacy

#### Summary

Tables 5 and 6 describe the patient activation and eHealth literacy scores based on the PAM-13 and eHEALS, respectively. Compared to participants without navigational needs, participants with navigational needs appear, on the whole, to be less activated (see Table 5) and have a lower level of eHealth literacy (see Table 6).

**Table 5:** Summary statistics: PAM-13 scores.

PAM-13 score (0.0-100.0)	No navigational needs (N=195)	Navigational needs (N=204)	Total (N=399)^a^
Mean (SD)	63.1 (12.5)	58.9 (13.3)	61.0 (13.1)
Median	60.6	58.1	58.1
Mode	55.6	63.1	55.6
Range	24.1-100.0	35.5-100.0	24.4-100.0

^a^Score could not be calculated for one participant due to invalid responses.

**Table 6:** Summary statistics: eHEALS scores.

eHEALS Score (8.0-40.0)	No navigational needs (N=195)	Navigational needs (N=205)	Total (N=400)
Mean (SD)	31.0 (4.1)	28.2 (4.2)	29.5 (4.3)
Median	31.0	28.0	30.0
Mode	32.0	32.0	32.0
Range	16.0-40.0	15.0-40.0	15.0-40.0

#### Associations

Correlations between PAM-13 and eHEALS scores revealed a positive, moderate association (*r*=.50, *P*<.001) (see Table 7). After inclusion of sex, age group (compared to the “55+” reference group), education (university vs no university level of education), and place of residence (major city vs rural) variables into a multivariate model, the only statistically significant predictor of PAM-13 scores was eHEALS scores (*P*<.001).

**Table 7:** PAM-13 score vs eHEALS score, and demographic variables.

		B	SE B	β
Constant		18.01	4.05	—
**Age groups**				
	18-24 (vs 55+)	-1.48	2.19	-.04
	25-34 (vs 55+)	-1.03	1.69	-.04
	35-44 (vs 55+)	-2.89	1.89	-.08
	45-54 (vs 55+)	-1.34	1.76	-.04
Female		-1.80	1.21	-.07
University education		1.45	1.21	.05
Living in major city		-0.85	1.27	-.03
eHEALS Score		1.53	0.13	0.51^a^
(R^2^=.27, Adj. R^2^=.25)				

^a^ *P*<.001

#### Reliability Tests

Internal consistency for the PAM-13 and eHEALS were assessed via Cronbach alpha for the test sample (n=400) and the retest sample (n=43). Relative test-retest reliability was assessed using ICC (3,1) to assess the overall test-retest reliability of the subset of the test sample (ie, n=43 from n=400) on retest. Results for the reliability tests indicate good-to-excellent internal consistency and excellent test-retest reliability (see Table 8).

**Table 8:** Reliability statistics for the PAM-13 and eHEALS.

		Cronbach α	ICC (3,1) (95% CI)
**eHEALS**			
	Test (n=400)	.87	N/A
	Test/Retest (n=43)	.92/.91	.79 (0.65-0.88)
**PAM-13**			
	Test (n=400)	.86	N/A
	Test/Retest (n=43)	.92/.88	.86 (0.75-0.92)

### Predictors of Navigational Needs

After inclusion of age, sex, level of education (university vs no university level education), place of residence (major city vs rural), the PAM-13 score, and the eHEALS score into a multivariate model, only age (*P*=.02)—specifically, the 45-54 age group (*P*=.048)—and the eHEALS score (*P*<.001) were statistically significant predictors of navigational needs (see Table 9).

Overall, the predictor variables (demographic variables, PAM-13, and eHEALS scores) used in this binary logistic regression analysis explained 18.7% of the variance in having navigational needs, measured using Nagelkerke’s R^2^[70].

**Table 9:** Predictors of navigational needs.

Predictors		Navigational needs (N=205)^a^	No navigational needs (N=195)^a^	OR (95% CI)	Adjusted OR (95% CI)
**Age group**					
	18-24	22 (10.7)	22 (11.3)	0.80 (0.39-1.67)	0.94 (0.42-2.11)
	25-34	71 (34.6)	48 (24.6)	1.17 (0.66-2.05)	1.54 (0.81-2.92)
	35-44	36 (17.6)	35 (17.9)	0.83 (0.44-1.56)	0.96 (0.48-1.96)
	45-54	30 (14.6)	52 (26.7)	0.46 (0.25-0.87)^b^	0.51 (0.26-0.99)^c^
	55+ (reference group for “age group” variable)	46 (22.4)	37 (19.0)	—	—
Female		123 (60.0)	122 (62.6)	0.90 (0.60-1.34)	0.98 (0.62-1.55)
University education		83 (41.1)	81 (41.5)	0.96 (0.64-1.43)	0.95 (0.60-1.50)
Living in major city		145 (40.5)	121 (62.1)	1.41 (0.93-2.15)	1.33 (0.83-2.15)
eHEALS score, mean (SD)		28.2 (4.2)	31.0 (4.1)	0.84 (0.80-0.89)^d^	0.83 (0.78-0.89)^d^
PAM-13 score, mean (SD)		58.9 (13.3)	63.1 (12.5)	0.98 (0.96-0.99)^e^	1.00 (0.98-1.02)

^a^Values presented as n (%) unless otherwise noted.

^b^ *P*=.02.

^c^ *P*=.048.

^d^ *P*<.001.

^e^ *P*=.002.

Based on adjusted odds ratios (adjusted OR), participants aged 45-54 years old were 0.51 times as likely to have navigational needs compared to participants aged 55 years and above. In other words, participants aged 45-54 years old were less likely to have navigational needs compared to participants aged 55 years and above. In addition, participants with a lower eHEALS score, that is, lower eHealth literacy, were more likely to have navigational needs.

## Discussion

### Principal Findings

Approximately half the population (51.3%, 95% CI 46.4-56.2) of consumers seeking Web-based health information and living with chronic health conditions was estimated to have navigational needs. These consumers reported at least some difficulty locating desired health information and indicated preferences for guidance to find desired health information on the Internet. While age and perceived eHealth literacy levels were associated with consumers’ navigational needs (Table 9), our study suggests that the majority of the population (91.3%), including consumers who did not report having difficulty locating desired Web-based health information, would still like some form of guidance. Given that approximately 75% of the total population in Australia reported having at least one chronic health condition [74], close to 75% of the Australian population has Internet access [5], and nearly 80% of Internet users use the Internet for health-related activities [5], it appears that a sizeable proportion of the total Australian population would likely be willing to receive some form of guidance in locating desired Web-based health information. A previous qualitative study [20] suggested health professionals could play a role in helping consumers locate desired Web-based health information. This and other types of assistance will be explored elsewhere using our current data.

Findings from this study suggest consumers with at least one chronic condition want to be more informed about their health, and consumers seek information as a way to help manage their conditions. These findings support literature on the use of the Internet as a mechanism by health consumers to assist self-management [6,7,11,26]. When comparing participants with and without navigational needs, this study found that participants with navigational needs appear to look for more types of Web-based health information and from a greater variety of sources. Thus, this study adds to existing literature by providing some descriptive characteristics about the Web-based HISB of consumers with navigational needs.

The majority of participants reported discussing Web-based health information with health professionals; the most common reason was to ascertain the opinions of health professionals on the retrieved health information. Only 10.3% of our participants indicated they use the Internet to find health information when disagreeing with advice from their health professionals. While not underestimating the proportion of these participants, this suggests consumers living with chronic health conditions predominantly use Web-based health information for reasons other than overriding advice given by health professionals. Collectively, these findings appear in line with studies [6-8] examining the role of the Internet in the consumer-health professional relationship, in that the Internet has the potential to better facilitate this relationship. However, when comparing participants with and without navigational needs, this study found that participants with navigational needs were less likely to be satisfied with their health professionals and more likely to not discuss information with their health professionals because they did not want to embarrass themselves in front of their health professionals (see Table 4). Thus, this study provides initial insight into aspects of HISB in consumers with navigational needs and suggests that health professionals may need to have conversations with consumers that greater encourage discussion of health information sought using the Internet.

In our study, when compared to participants without navigational needs, participants with navigational needs appeared to have lower levels of patient activation and eHealth literacy (Tables 5 and 6). However, as established earlier, participants with navigational needs sought more types of health information from a greater variety of Web-based sources. Furthermore, participants with navigational needs were more likely to report that they discussed information sought using the Internet with their health professionals for the purpose of obtaining more information (Table 3). Such findings suggest that, despite have a seemingly greater desire to obtain information, participants with navigational needs are less able to find such information (lower eHealth literacy) and are less confident in their searching abilities (lower patient activation). This reinforces the need to provide assistance to consumers with navigational needs and provides further justification that more research needs to be conducted to address navigational needs.

Further to our use of the PAM-13 and eHEALS measures, our data revealed a moderate but statistically significant correlation between the PAM-13 and eHEALS (*r*=.50, *P*<.001), supporting a relationship between patient activation and perceived eHealth literacy, as well as confirming other studies [55,56]. These findings extend the literature in that patient activation appears to be a prominent concept in the context of eHealth literacy and suggests this association is present even after accounting for demographic variables of age, sex, level of education, and place of residence (major cities versus rural). While self-perceived eHealth literacy refers to individuals’ self-perceived abilities to obtain and utilize Web-based health information for the purpose of self-management [52], patient activation refers to individuals’ self-belief in their behavioral repertoires, abilities, and knowledge pertaining to self-management [54]. Given the apparent overlap in these two concepts, health information consumers who self-identify as being motivated and having the ability and knowledge to self-manage their conditions could also be assumed to be more adept at utilizing the Internet for self-management purposes.

The validity and reliability of the eHEALS and PAM-13 have been well established [54,60-68]. Our internal consistency and test-retest reliability analysis confirmed the reliability of both instruments in the current sample. Given that reliability is a prerequisite for validity [75], and the pre-establishment of validity in these two measures, these measures are likely to also be valid in our sample. By using these two measures as proxies for key concepts in predicting navigational needs, we believe our conclusions regarding the predictors of navigational needs are empirically justified.

### Strengths and Limitations

A key strength of this study lies in our overall approach to developing the questionnaire. Specifically, the use of attention filters and a duration filter helped ensure that our participants provided complete and valid responses. The incorporation of two scales (PAM-13 and eHEALS) with prior evidence of validity and reliability allowed for trustworthy conclusions to be drawn from the data. The use of forced responses minimized potential for missing data; only one participant’s PAM-13 score could not be calculated from having selected several “Not Applicable” options within the scale. The use of questions and response items pertaining to Web-based HISB and navigational needs, based on a qualitative study conducted on a similar target population [20], provided initial empirical validation. This means the characteristics of consumers’ Web-based HISB and navigational needs from our study may more accurately reflect the target population, compared to questionnaires where the items had not been created from the consumer perspective. Furthermore, our use of randomization of response items perceivably mitigated response bias.

Various steps ensured our questions were easily comprehended by our participants: inviting participants to comment on comprehension in our pilot survey, face validity checks by our research team, and the use of the Flesch-Kincaid Grade level test. The readability score of 8.0 indicates participants who have completed at least the 8th grade of formal education would be able to comprehend the questionnaire [69]. Based on the demographics of our participants, this suggests that 99.5% of our participants (398/400 participants) would have been able to comprehend the questions and response items in our questionnaire.

While we requested Qualtrics to gather a representative sample of the Australian population, information pertaining to their sampling technique in doing so was not disclosed. As established earlier, there is a lack of data on population demographics in the context of Australian Internet use, limiting our ability to compare this sample with national demographic data. Furthermore, a representative sample does not necessarily translate to a random sample. Given our sample size calculation for prevalence studies assumes random sampling technique, the level of precision for our study cannot be accurately determined, and external validity cannot be assured. However, based on a report [76] from the Australian Institute of Health and Welfare (AIHW), the population prevalence versus the prevalence in our sample is comparable for cardiovascular diseases (22% vs 29.8%) and mental health conditions (20% vs 25.5%)—two of the three most commonly reported conditions in our sample (Table 2). This comparison, however, does not take into consideration variation in the prevalence of such conditions between Internet users and non-users. Furthermore, our sample is of consumers with chronic health conditions, whereas the AIHW report [76] expresses prevalence as a proportion of the entire Australian population. Nevertheless, our sample size was sufficient for the required analyses, and given the moderately large sample size and the diverse demographic characteristics of our participants, it appears our findings can be applied to a wider population.

A further limitation to our study was the use of multiple-response, multiple-choice questions for our Web-based HISB domain to generate a comprehensive description of this domain. The permutations of options meant the data were only reported descriptively; a much larger sample would be required to facilitate comparisons between cohorts of respondents. Similarly, test-retest reliability of such multiple-response, multiple-choice questions could not be determined.

This study did not determine the device(s) or platform(s) used to access Web-based health information. The availability of mobile-friendly versions of certain websites should improve access to Web-based health information [77]. In addition, our questionnaire did not explicitly explore social media as a health information source. These responses were elicited via an “Other” option (Table 5), but not to the extent suggested in literature reporting social network sites are becoming popular sources of general information for many users [42]. While such social network sites are reportedly less popular for people with chronic health conditions in the United States [42], their use is reportedly increasing [78].

### Further Research

Despite the perception of the health professional as the most trusted source of Web-based health information [79], our study suggests that their role in guiding consumers to Web-based health information appears underutilized by consumers. We therefore recommend further investigation into why this role is underutilized and believe that both consumer and health professional perspectives should be explored. Once more in-depth understanding is attained, further research could explore the current roles of various health professions and investigate pragmatic ways that navigational guidance can be provided. While initiatives such as the use of social networking technologies by health professionals to provide guidance [80] and “information prescriptions” [81-85] have been implemented, to our knowledge, these initiatives have not considered consumers’ navigational needs, and this represents a topic for future development.

While age and perceived eHealth literacy levels were found to be statistically significant predictors of navigational needs, the variables included in our multivariate binary logistic regression model explain only 18.7% of the variance in a consumer being identified with navigational needs. Other variables associated with the navigational needs of consumers remain unexplored, and these may inform individualized approaches to supporting navigational needs. We therefore recommend further investigation into identifying additional predictors of navigational needs.

One could expect consumers with higher levels of perceived eHealth literacy would be less likely to have navigational needs. Indeed, this was the case in our study. Our study also identified a significantly lower likelihood of navigational needs in participants aged 45-54 years, compared to those aged 55 years and above. Further investigation is recommended to determine characteristics of this middle-aged group, why this specific age group was less likely to have navigational needs compared to those 55 years and above, and suitable interventions to meet their needs.

A positive correlation was found between patient activation and eHealth literacy, albeit moderate. Until empirical data can better account for the variance in the relationship, future interventions aimed to address either patient activation or eHealth literacy should retain both constructs. Finally, given our universal approach to exploring Web-based HISB of health information consumers with a variety of chronic health conditions, future studies that focus on specific chronic health conditions can compare their findings against this study to determine commonalities and variations between and across chronic conditions.

### Conclusions

This study highlights the proportion of people with chronic health conditions who use the Internet and who have navigational needs, and reports that a majority of this population would want help locating desired Web-based health information. While we identified a number of associations that help identify individuals who would benefit from guidance in navigating Web-based health information, given that the majority of the population would want assistance, more universal approaches may be valuable to help all consumers locate desired Web-based health information.

## Appendix

Multimedia Appendix 1 Reported chronic health conditions (N=400).

Multimedia Appendix 2 Health information sought (N=400).

Multimedia Appendix 3 Where Web-based health information is usually sought (N=400).

Multimedia Appendix 4 Action(s) taken upon finding Web-based health information (N=400).

## Abbreviations

AIHW: Australian Institute of Health and Welfare
eHEALS: eHealth Literacy Scale
HISB: health information-seeking behaviors
ICC: intraclass correlation coefficient
PAM-13: 13-item Patient Activation Measure
