# Supplementary material for: Consumer Use of “Dr Google”: A Survey on Health Information-Seeking Behaviors and Navigational Needs
Source: J Med Internet Res. 2015 Dec 29;17(12):e288. doi: 10.2196/jmir.4345 (PMC4710847; doi:10.2196/jmir.4345)
Supplement: Multimedia Appendix 1 [file jmir_v17i12e288_app1.pdf]

Multimedia Appendix 1. Reported chronic health conditions (N=400).

Health condition	No navigational needs (N=195) n (%)[a]	Navigational needs (N=205) n (%)[a]	Total n (%)[a]
Heart and vascular condition (eg, high blood pressure, stroke, heart failure, angina, high cholesterol)	58 (29.7)	61 (29.8)	119 (29.8)
Skin condition (eg, eczema, psoriasis)	45 (23.1)	59 (28.8)	104 (26.0)
Mental health (eg, depression)	50 (25.6)	52 (25.4)	102 (25.5)
Lung condition (eg, asthma, COPD, emphysema)	50 (25.6)	41 (20.1)	91 (22.8)
Stomach or bowel condition (eg, irritable bowel syndrome, Crohn's disease, ulcerative colitis, reflux)	44 (22.6)	37 (18.0)	81 (20.3)
Arthritis and/or osteoporosis	39 (20.0)	35 (17.1)	74 (18.5)
Diabetes (Type 1 or Type 2)	24 (12.3)	28 (13.7)	52 (13.0)
Thyroid condition	14 (7.2)	17 (8.3)	31 (7.8)
Kidney disease	7 (3.6)	3 (1.5)	10 (2.5)
Cancer – not related to any of the above conditions	6 (3.1)	13 (6.3)	19 (4.8)
Other	43 (22.1)	54 (26.3)	97 (24.3)

[a]Respondents could select multiple options; percentages do not total 100%.
