# Supplementary material for: Consumer Use of “Dr Google”: A Survey on Health Information-Seeking Behaviors and Navigational Needs
Source: J Med Internet Res. 2015 Dec 29;17(12):e288. doi: 10.2196/jmir.4345 (PMC4710847; doi:10.2196/jmir.4345)
Supplement: Multimedia Appendix 2 [file jmir_v17i12e288_app2.pdf]

Multimedia Appendix 2. Health information sought (N=400).

Category of health information	No navigational needs (N=195) n (%)[a]	Navigational needs (N=205) n (%)[a]	Total n (%)[a]
Information about medical conditions	173 (88.7)	184 (89.8)	357 (89.3)
Information about medicines or medical devices	136 (69.7)	139 (67.8)	275 (68.8)
Information about diets	109 (55.9)	123 (60.0)	232 (58.0)
Medical terms or jargon	110 (56.4)	121 (59.0)	231 (57.8)
Information about natural products (eg, herbal products, vitamin supplements)	107 (54.9)	124 (60.5)	231 (57.8)
Information about health professionals, clinics or hospitals	105 (53.8)	118 (57.6)	223 (55.8)
Information about exercise(s)	98 (50.3)	123 (60.0)	221 (55.3)
Information about associations or societies (eg, Diabetes Association, Cancer Council)	63 (32.3)	84 (41.0)	147 (36.8)
Other	10 (5.1)	8 (3.9)	18 (4.5)

[a]Respondents could select multiple options; percentages do not total 100%.
