# Supplementary material for: Consumer Use of “Dr Google”: A Survey on Health Information-Seeking Behaviors and Navigational Needs
Source: J Med Internet Res. 2015 Dec 29;17(12):e288. doi: 10.2196/jmir.4345 (PMC4710847; doi:10.2196/jmir.4345)
Supplement: Multimedia Appendix 3 [file jmir_v17i12e288_app3.pdf]

Multimedia Appendix 3. Where web-based health information is usually sought (N=400).

Source of web-based information	No navigational needs (N=195) n (%)[a]	Navigational needs (N=205) n (%)[a]	Total n (%)[a]
Search engines (eg, Google, Yahoo, Bing)	170 (87.2)	174 (84.9)	344 (86.0)
General health websites (eg, BetterHealth, Web MD)	110 (56.4)	120 (58.5)	230 (57.5)
Association websites specific to my medical condition(s) (eg, Diabetes Association, Cancer Council)	87 (44.6)	114 (55.6)	201 (50.3)
Wikipedia	71 (36.4)	80 (39.0)	151 (37.8)
Websites recommended by health professionals	68 (34.9)	83 (40.5)	151 (37.8)
Private health insurer websites (eg, MediBank, Bupa)	26 (13.3)	47 (22.9)	73 (18.3)
Websites recommended by people on discussion forums	35 (17.9)	37 (18.0)	72 (18.0)
Research databases (eg, PubMed, Cochrane, CINAHL)	37 (19.0)	35 (17.1)	72 (18.0)
eNewsletters or emails that you have signed up for	25 (12.8)	24 (11.7)	49 (12.3)
Other	7 (3.6)	6 (2.9)	13 (3.3)

[a]Respondents could select multiple options; percentages do not total 100%.
