# Supplementary material for: Consumer Use of “Dr Google”: A Survey on Health Information-Seeking Behaviors and Navigational Needs
Source: J Med Internet Res. 2015 Dec 29;17(12):e288. doi: 10.2196/jmir.4345 (PMC4710847; doi:10.2196/jmir.4345)
Supplement: Multimedia Appendix 4 [file jmir_v17i12e288_app4.pdf]

Multimedia Appendix 4. Action(s) taken upon finding web-based health information (N=400).

Action	No Navigational Needs (N=195) n (%)[a]	Navigational Needs (N=205) n (%)[a]	Total n (%)[a]
I use the information to decide whether I need to see a health professional	110 (56.4)	123 (60.0)	233 (58.3)
I use the information to change my diet or exercise	95 (48.7)	113 (55.1)	208 (52.0)
I discuss the information with a health professional	97 (49.7)	110 (53.7)	207 (51.8)
I use the information to decide whether to buy a medicine or natural product	80 (41.0)	94 (45.9)	174 (43.5)
Other	16 (8.2)	9 (4.4)	25 (6.3)

[a]Respondents could select multiple options; percentages do not total 100%.
